# Draft Genome Sequence of an Oxalate-Degrading Strain of *Clostridium sporogenes* from the Gastrointestinal Tract of the White-Throated Woodrat (*Neotoma albigula*)

**DOI:** 10.1128/genomeA.00392-16

**Published:** 2016-05-19

**Authors:** Kelly F. Oakeson, Aaron Miller, Colin Dale, Denise Dearing

**Affiliations:** a University of Utah, Department of Biology, Salt Lake City, Utah, USA; b The Cleveland Clinic, Cleveland, Ohio, USA

## Abstract

The gastrointestinal tract of the white-throated woodrat *Neotoma albigula* harbors a diverse microbial population that functions in the degradation of ingested plant secondary compounds. Here, we present the draft genome sequence and annotation of *Clostridium sporogenes* strain 8-O, a novel oxalate-degrading bacterium isolated from the feces of *N. albigula*.

## GENOME ANNOUNCEMENT

In some habitats, *Neotoma albigula* consumes a diet consisting primarily of *Opuntia cactus* which is rich in oxalate (1). Like all mammals, *N. albigula* is not capable of metabolizing oxalate, and relies on bacterial populations in their gut to degrade oxalate. Previous work has demonstrated that *N. albigula* houses multiple oxalate-degrading taxa throughout its gastrointestinal tract, and many of these oxalate-degrading taxa have been cultured and their oxalate-degrading capabilities quantified (2). Bacterial isolation and oxalate-degrading capabilities were previously described in Miller et al. (2). In brief, gut contents of the foregut, stomach, small intestine, cecum, and large intestines were collected along with the feces isolated from *N. albigula* and were serially diluted in sterile deionized water. Dilutions were then plated onto brain heart infusion (BHI) agar supplemented with 20 mM sodium oxalate and 1 g/L calcium chloride. Cultures were incubated at 37°C for 48 h under anaerobic conditions (Anaeropack system, Mitsubishi Gas Chemical Company). Individual colonies were selected for further enrichment by the presence of a zone of clearance around the colony, indicating the degradation of oxalate. To evaluate *in-vitro* oxalate degradation, isolates were inoculated into BHI broth supplemented with 20 mM sodium oxalate and incubated for 48 h. The oxalate concentration of the medium after the incubation period was then determined by titration. Isolates that showed significant oxalate degradation were identified by 16 s sequencing in comparison to the Ribosomal Database Project (RDP). The isolate that showed the highest oxalate degradation efficiency was 99% similar to *Clostridium sporogenes* strain CL2 (GenBank accession number JF836013.1) and was selected for whole-genome sequencing. Genomic DNA isolated from a liquid culture of *C. sporogenes* using Qiagen DNeasy blood and tissue kit (Qiagen, Valencia, CA) and submitted to the High Throughput Genomics Core Facility at The Huntsman Cancer Institute (Salt Lake City, UT) for library preparation and sequencing. Library construction was performed using the Illumina TruSeq nano DNA preparation kit (catalog number FC-121-9010, Illumina, Inc., San Diego, CA) generating a library with a mean insert size of 550 bp. The resulting library was sequenced with the Illumina MiSeq platform generating 19,515,731 paired-end reads of 250 bp. Paired-end reads were quality filtered and trimmed using Trimmomatic (3) and any overlapping pair-end reads were joined using PEAR (4). The merged overlapping reads, along with all nonoverlapping paired-end reads were then assembled using the SPAdes assembler release 3.5.0 (5). The resulting assembly consisted of 19 scaffolds of 28 contigs and 4,088,089 bp of sequence with a G+C content of 27.75%. The assembled genomic sequences were submitted to the National Center for Biotechnology Information (NCBI) Prokaryotic Genomes Automatic Annotation Pipeline (PGAAP) (6) for annotation. The resulting annotation consists of 3,639 coding sequences (CDSs), 120 pseudogenes, 77 tRNAs, 4 noncoding RNAs (ncRNAs), and 27 rRNAs, many of which are partial sequences located at the end of scaffolds. Inspection of the annotation revealed a single gene involved in oxalate metabolism, an oxalate/formate anti-porter.

### Nucleotide sequence accession numbers. 

This whole-genome shotgun project has been deposited at DDBJ/ENA/GenBank under the accession number LUAU00000000. The version described in this paper is version LUAU01000000.
